# Which test for crossing survival curves? A user’s guideline

**DOI:** 10.1186/s12874-022-01520-0

**Published:** 2022-01-30

**Authors:** Ina Dormuth, Tiantian Liu, Jin Xu, Menggang Yu, Markus Pauly, Marc Ditzhaus

**Affiliations:** 1 TU Dortmund University, Joseph-von-Fraunhofer-Straße 2-4, 44221 Dortmund, Germany; 2 Technion – Israel Institute of Technology, Haifa, Israel; 3 East China Normal University, Shanghai, China; 4 University of Wisconsin-Madison, Madison, USA

**Keywords:** Survival analysis, Time-to-event outcome, Crossing, Non-proportional hazards, Oncology, Log-rank test, Restricted-mean survival

## Abstract

**Background:**

The exchange of knowledge between statisticians developing new methodology and clinicians, reviewers or authors applying them is fundamental. This is specifically true for clinical trials with time-to-event endpoints. Thereby, one of the most commonly arising questions is that of equal survival distributions in two-armed trial. The log-rank test is still the gold-standard to infer this question. However, in case of non-proportional hazards, its power can become poor and multiple extensions have been developed to overcome this issue. We aim to facilitate the choice of a test for the detection of survival differences in the case of crossing hazards.

**Methods:**

We restricted the review to the most recent two-armed clinical oncology trials with crossing survival curves. Each data set was reconstructed using a state-of-the-art reconstruction algorithm. To ensure reproduction quality, only publications with published number at risk at multiple time points, sufficient printing quality and a non-informative censoring pattern were included. This article depicts the *p*-values of the log-rank and Peto-Peto test as references and compares them with nine different tests developed for detection of survival differences in the presence of non-proportional or crossing hazards.

**Results:**

We reviewed 1400 recent phase III clinical oncology trials and selected fifteen studies that met our eligibility criteria for data reconstruction. After including further three individual patient data sets, for nine out of eighteen studies significant differences in survival were found using the investigated tests. An important point that reviewers should pay attention to is that 28% of the studies with published survival curves did not report the number at risk. This makes reconstruction and plausibility checks almost impossible.

**Conclusions:**

The evaluation shows that inference methods constructed to detect differences in survival in presence of non-proportional hazards are beneficial and help to provide guidance in choosing a sensible alternative to the standard log-rank test.

**Supplementary Information:**

The online version contains supplementary material available at 10.1186/s12874-022-01520-0.

## Background

Time-to-event studies are the paramount studies in clinical practice. Typical examples are two-armed trials providing a reliable comparison of the efficacy and safety of two treatments. Statistical methods that infer a potential difference in survival are of fundamental importance [1]. Among methods designed to compare the overall survival of two groups, the log-rank test (LR) is still the most used [2]. Beyond a certain resistance to statistical innovations [3], there is also a theoretical reason: The LR is optimal in case of proportional hazards (PH) [4]. In other words, if the hazard functions of the two groups are proportional, the LR is the most powerful method to detect differences between them. However, this changes completely for other kinds of hazard patterns, in particular for crossing hazards and the rejection rates of the LR drop significantly. The alarming observation of Kristiansen [5], who reviewed 175 studies in five renowned journals, is that the LR was applied in 70% of the cases despite crossing survival curves. These crossings can occur e.g. in oncology when comparing tumor dissection versus radiation strategies due to different time-dependent effects.

Consequently, several methods have been and are still proposed to tackle non-PH situations. However, due to the speed of research and the number of new methods, the exchange of knowledge is a challenge. Therefore, Ananthakrishnan et al. [6] recently provided a critical review on methods in the presence of possible non-PHs and their limitations and advantages. While they give detailed information regarding the assumptions and the context, they do not provide any numerical evaluation of the methods. We include here state of the art tests with the aim of providing biostatisticians, physicians and reviewers with a condensed overview of suitable methods for non-PH settings that are implemented in the open statistical software R. These methods not only show good results in various simulation studies but also on real data.

## Methods

There are several papers that develop alternatives to the LR in case of non-PH or even crossing hazards. Treating them all would go far beyond the scope of this work. Hence, we focused our comparisons on standard methods that performed well in other simulation studies and more recent ones that were not yet included in extensive evaluations. Here, all analyses are conducted using the free and open-source software R [7] (except for the test introduced by Royston [8]).

Fortunately, the paper by Li et al. [9] already provides a review on methods for crossing hazards up to 2014. Based on extensive simulation studies they recommend two procedures: First, Neyman’s smooth test proposed by Kraus [10]. This test is not considered further since the corresponding R package was removed recently. Second, a two-stage procedure (2ST) that is based on the LR and a crossing-hazards test is proposed (see the Supplement for more details.). The test is described by Qiu and Sheng [11] and implemented in the R package *TSHRC* [12].

Further methods have been developed since 2014. We have included the most relevant ones into our study. For example, Gorfine et al. [13] presented two omnibus permutation tests based on a sample space partition, which showed promising results in non-PH situations. These are either based on test statistics of Pearson’s chi square (KONP chi) or likelihood-ratio type (KONP llr) and are available in the R package *KONPsurv* [14]. They compared their new approach with the well-established test of Yang and Prentice [15], which belongs to the class of weighted log-rank tests and employs adaptive weights. Since Gorfine et al. [13] could show in simulations that their new tests are more powerful in the studied non-PH settings, the Yang and Prentice test is not included in our comparison. Another idea starts with the class of weighted LR. This class is long known and includes the LR as well as the common Peto-Peto test (PP). Recently, a flexible combination of several weighted LRs into one test procedure was proposed [16–18]. It is based upon a combination of alternatives and carried out as a permutation procedure. Recently, it has been implemented in the R package *mdir.log-rank* [19]. The multiple-direction log-rank test (mdir) combines several weighted log-rank tests into one joint Wald-type statistic, which can be interpreted as a projection on a large alternative space spanned by pre-chosen weights. The latter ensures that mdir has not only a reasonable power in the directions of the chosen weights (e.g. for PHs or a specific crossing curve situation) but also in the directions of any linear combination of the pre-chosen weights. Moreover, the weights are allowed to be data-dependent. Another approach that combines multiple weighted log-rank tests is the MaxCombo test (MaxCombo). Different to mdir, the final test statistic is the maximum over standardized weighted LR tests [20]. We used the same list of weights as proposed in the description of the *nphsim* package [21]. We refer to the supplement for specific as well as technical details on all methods. Besides HR, the restricted mean survival time (RMST) can be used to quantify the difference between two survival curves [22]. It describes the mean event-free survival time up to a pre-defined time point *τ*. Hypothesis tests constructed using the RMST examine whether the RMST difference between groups is zero. This test is also valid to test equality of two survival functions, since equal survival functions imply equal RMST. Unfortunately, it is possible to observe situations where the RMSTs are equal but the survival functions are not. This has to be kept in mind while using RMST-based tests. We consider three RMST-based proposals: The first two utilize the group-wise RMST differences as test statistic and either calculate *p*-values based on resampling (RMST1) or obtained using asymptotic theory (RMST2) [23, 24]. The former is provided by the R package *surv2sample* [25] while the latter can be computed with the function *rmst2* in *survRM2* [26]. Eventually, Royston and Parmar [27, 28] propagate a test combining a Cox test and a permutation-based RMST test (coxRMST). The test by Royston and Parmar is only available in STATA using the *stctest* function. Finally, we consider a test based on an integrated *L*_1_-distance of the two Kaplan-Meier curves as test statistic. It can be interpreted as the area between curves (ABC) and was introduced in Liu et al. [29]. It has not been implemented in R yet and was thus coded by ourselves according to the author’s descriptions. The code can be found in the supplements.

A detailed description of all eleven tests and corresponding test statistics can be found in the Supplement. Furthermore, a simple example in R is given in the Supplement. Below we will compare them based upon different studies. To this end, we reconstruct data from published Kaplan-Meier curves using the algorithm developed by Guyot et al. [30] and deriving the data from the curves with the freely available *Webplotdigitizer* [31].

## Results

### Eligibility screening and data extraction

**Fig. 1:**
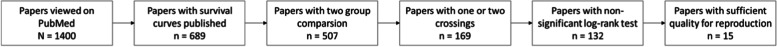
Flow chart of papers under consideration

Our study was motivated by the work of Matabuena and Padilla [32] which includes three oncology studies with crossing Kaplan Meier (KM) curves. We subsequently performed a PubMed screening of recent oncology studies with similar patterns. To ensure these patterns, the search matched *((Phase 3) OR (phase III)) OR (Kaplan-Meier) OR (Kaplan Meier))* for Cancer and Humans were used. To categorize them, multiple criteria listed in Fig. 1 were defined to identify relevant studies on PubMed. 1400 of the most recent papers (status from Oct 5, 2020) on clinical oncology were searched for crossing survival curves with published number at risk at multiple time points. More details can be found in eTable 1 in the online Supplement. The executed LR test had to be non-significant and the two arms should only cross one or two times. To ensure a good reconstructibility, a sufficient number of events and high quality of the curves as well as non-informative censoring over time were required. In the end, the reconstruction algorithm of Guyot et al. [30] was applied to fifteen publications that met these requirements and the three studies discussed in the paper of Matabuena and Padilla [32]. Beyond insufficient information (e.g., almost 30% of the publications did not report the number at risks) another reason for the final small number of publications can be publication bias since non-significant results are less often reported.

### Data reconstruction

The individual patient data from the three studies found in Matabuena and Padilla [32] and the fifteen other studies under consideration [33–50] were reconstructed using the algorithm introduced by Guyot et al. [30]. To assess the quality of reconstruction, the reported key statistics (median survival and HR with confidence interval) published in each paper were recalculated and compared to the original values (see Table 1).

**Table 1:** Assessment of data reconstruction quality

Publication	MS G1	MS G2	HR [CI]
Bang et al. (2020) [37]	5.80 (5.88)	4.30 (4.44)	0.83 [0.53, 1.31] (0.82 [0.52, 1.29])
Becker et al. (2020) [39]	not defined	6.00 (6.21)	5.50 (5.51)
Bellmunt et al. (2017) [45]	3.30 (3.24)	2.10 (2.08)	0.98 [0.81, 1.19] (0.93 [0.77, 1.13])
Cortes et al. (2019) [46]	4.90 (4.94)	4.70 (4.72)	0.63 (0.62)
Ferris et al. (2016) [41]	2.00 (2.02)	2.30 (2.29)	0.89 [0.70,1.13] (0.89 [0.70,1.14])
Fradet et al. (2019) [47]	3.30 (3.35)	2.10 (2.16)	0.96 [0.79, 1.16] (0.92 [0.77, 1.11])
Godfrey et al. (2018) [36]	–	–	1.40 [0.54, 3.61] (1.40 [0.53, 3.69])
Golan et al. (2019) [38]	18.90 (18.90)	18.10 (18.10)	0.91 [0.56, 1.46] (0.88 [0.55, 1.42])
Hammel et al. (2019) [35]	21.20 (21.36)	6.00 (5.93)	0.72 [0.41, 1.27] (0.72 [0.42, 1.24])
Jones et al. (2020) [34]	26.00 (26.00)	20.0 (18.80)	0.59 [0.34, 1.05] (0.58 [0.33, 1.02])
Jones et al. (2018) [33]	15.10 (15.08)	8.10 (8.02)	0.72 [0.45, 1.17] (0.71 [0.44, 1.15])
Kotani et al. (2019) [48]	8.60 (8.62)	8.00 (8.02)	0.74 [0.48, 1.14] (0.72 [0.47, 1.11])
Kreuzer et al. (2020) [50]	19.40 (19.40)	20.90 (21.30)	1.22 [0.60, 2.47] (1.26 [0.62, 2.56])
Lu et al. (2018) [40]	4.63 (4.68)	4.23 (4.33)	0.78 [0.60, 1.00] (0.74 [0.55, 1.01])
Malone et al. (2020) [49]	–	–	0.66 [0.41, 1.07] (0.68 [0.42, 1.10])
Motzer et al. (2015) [42]	4.60 (4.46)	4.40 (4.07)	0.88 [0.75, 1.03] (0.87 [0.98, 1.34])
Mukai et al. (2019) [44]	27.90 (27.90)	16.60 (16.60)	0.55 [0.23, 1.29] (0.55 [0.23, 1.29])
Toxopeus et al. (2018) [43]	–	–	1.02 [0.75, 1.39] (1.01 [0.75, 1.39])

Quality of data reconstruction regarding the published median survival (MS) in group 1 and 2 (G1 and G2), the hazard ratio (HR) with 95% confidence intervals (CI). For each study the published statistics are given with the corresponding statistics of the reconstructed data in parentheses. Three studies did not report MS (−) and two did not provide confidence intervals

### Comparison of tests for proportional hazards and crossing hazards

The reconstructed individual patient data were then used to compare the different testing approaches. For all resampling-based methods, the number of iterations was set to 5000 and for all RMST procedures the parameter *τ* was set to 90% of the minimum of the largest censored or uncensored time among the arms [51]. The results are listed in Table 2.

**Table 2:** *P*-values of the different tests applied to the reconstructed individual patient data of each publication

Publication	LR	PP	RMST1	RMST2	coxRMST	KONP_chi	KONP_llr	Mdir	2ST	ABC	MaxCombo
Bang et al. (2020) [37]	0.37	0.07	0.11	0.12	0.11	0.14	0.15	**0.03**	0.06	0.13	0.1
Becker et al. (2020) [39]	0.09	0.47	0.22	0.22	**0.02**	0.14	0.09	**0.02**	0.27	0.15	**0.04**
Bellmunt et al. (2017) [45]	0.49	**0.03**	0.38	0.38	**0.003**	**< 0.001**	**< 0.001**	**< 0.001**	**0.03**	**0.002**	**< 0.001**
Cortes et al. (2019) [46]	0.19	0.24	0.23	0.24	0.28	0.40	0.36	0.41	0.87	0.29	0.56
Ferris et al. (2016) [41]	0.33	0.84	0.23	0.23	0.25	**0.01**	**0.009**	**0.02**	**0.04**	**0.03**	**< 0.001**
Fradet et al.(2019) [47]	0.40	**0.02**	**0.04**	**0.04**	**0.009**	**< 0.001**	**< 0.001**	**< 0.001**	**0.03**	**0.001**	**< 0.001**
Godfrey et al. (2018) [36]	0.49	0.48	0.58	0.59	0.63	0.18	0.20	0.75	0.90	0.44	0.74
Golan et al. (2019) [38]	0.61	0.78	9.74	0.75	0.50	0.58	0.59	0.61	0.22	0.61	0.66
Hammel et al. (2019) [35]	0.22	0.35	0.62	0.62	0.33	0.19	0.19	0.16	0.27	0.38	0.09
Jones et al. (2020) [34]	0.05^a^	0.11	0.14	0.14	0.07	**0.02**	**0.02**	0.12	0.41	0.11	**0.04**
Jones et al. (2018) [33]	0.17	**0.03**	**0.02**	**0.02**	**0.05**	**0.03**	**0.04**	**0.009**	**0.05**	**0.03**	0.05^a^
Kotani et al. (2019) [48]	0.14	0.24	0.38	0.38	0.20	0.48	0.48	0.28	0.45	0.45	0.17
Kreuzer et al. (2020) [50]	0.53	0.25	0.07	0.08	0.17	0.28	0.28	0.10	0.10	0.07	0.27
Lu et al. (2018) [40]	0.06	**0.007**	**0.02**	**0.02**	**0.02**	0.07	0.07	**0.007**	**0.04**	**0.01**	**0.01**
Malone et al. (2020) [49]	0.11	0.12	0.13	0.13	0.17	0.08	0.09	0.28	0.57	0.12	0.22
Motzer et al. (2015) [42]	0.07	0.51	0.13	0.13	0.11	**0.03**	**0.03**	**0.01**	0.26	0.08	**< 0.001**
Mukai et al. (2019) [44]	0.17	0.22	0.15	0.17	0.26	0.22	0.25	0.33	0.53	0.16	0.44
Toxopeus et al. (2018) [43]	0.91	0.84	0.75	0.75	0.35	0.37	0.36	0.15	0.11	0.56	0.34

^a^Only 0.05 due to rounding down

Bold values indicate *p*-values smaller than the 5% type-I error level

It can be observed that the LR test never succeeds to reject the null hypothesis of equal survival in both groups at the 5% level. This leads to the exact same conclusion as in the eighteen published studies. The PP is designed to find early differences [52]. It succeeds in revealing an inequality in survival for four of the eighteen studies under consideration [33, 40, 45, 47]. Let us next consider the three RMST tests. These do not rely on the assumption of PHs but are also not specifically designed to detect crossings [53]. The resampling-based (RMST1) and the distribution-based version (RMST2) reject the null hypothesis in three cases [33, 34, 40], while the combined test (coxRMST) rejects the null hypothesis in five cases [33, 39, 40, 45, 47]. These findings support the analyses of Royston et al. [54]. The six remaining tests are all omnibus tests with different properties. The two tests by Gorfine et al. [13]. (KONP chi and KONP llr) find differences in survival in the same six cases [33, 34, 41, 42, 45, 47]. The omnibus test by Ditzhaus and Friedrich [17] (mdir) can reject the null hypothesis in eight out of eighteen cases [33, 37, 39–41, 45, 47]. The two-stage procedure (2ST) detects differences in five out of eighteen data sets [33, 40, 41, 45, 47]. The ABC has significant results for the same five studies as the two-stage test [33, 40, 41, 45, 47]. The MaxCombo test leads to *p*-values smaller than 0.05 for seven of the eighteen data sets [34, 39–42, 45, 47]. In these specific data examples, the test by Ditzhaus and Friedrich [14] is the test that detects the most differences. These results are consistent with those of Li et al. [9], Gorfine et al. [13] and Royston and Parmar [28] who also indicated that omnibus tests have greater power when deviating from the proportional hazards assumption. Evaluation of the methods’ performance under PHs reveals that almost all of the approaches reject the null hypothesis when the LR does (for details see the Supplement). In future simulation studies, the performance of the tests and their extensions to multi-arm settings will be further evaluated [13, 55–57].

## Discussion

To assess efficacy of two treatments the LR is generally regarded as the gold standard. The LR is optimal in terms of power under the PH assumption but can lose sufficient power in non-PH situations. The results of our PubMed analysis, however, show that there are many situations, where the LR is used in case of non-PH. At the same time, several alternatives are presented, which succeed to detect differences where the LR fails. The majority of these tests are available in statistical software (R). Hence, their execution is almost as user-friendly as calculating the LR. To furtherfacilitate their application, we provide minimal examples on how to use the implemented R functions in the supplement.

To exemplify the different implications, we reconstructed individual patient data from eighteen recent oncology trials that met the eligibility criteria of our analysis. In particular, high quality KM plots with sufficient information were necessary for the reconstruction algorithm. Based on these eighteen studies we compared the test decisions of eleven different testing procedures. It turns out, that the LR alternatives can exhibit power to identify differences between groups. Omnibus approaches, which have high power against several alternatives (such as PH and crossings in case of the mdir test), turned out to be particularly suitable for this purpose (see the Supplement for additional information regarding PH performance).

### Limitations

One of the main limitations of this kind of study is the dependence on the selection of data sets. To make a clear statement regarding the quality of the individual procedures in a direct comparison, extensive simulation studies are necessary. These are part of our own ongoing research. Nevertheless, it can be said that the LR cannot reject the null hypothesis in real situations involving non-proportional hazards included in this paper, while various omnibus tests are able to do so. Furthermore, the data used here are reconstructed individual patient data and thus does not have the same quality as the original data. While many properties of the data such as non-proportionality are conserved, the biggest reconstruction issue is the assumption of uniformly distributed censoring times. However, the assessment of the reconstruction quality turned out to be very satisfying.

### Recommendations for reviewers

Regarding the insights of our investigation, attention in the reviewing process of study reports should be paid to

1. the appropriate choice of the statistical method. Especially when the PH assumption cannot be justified in advance, e.g. by a preliminary study, alternatives to the LR should be considered. Due to multiplicity issues, we do not advocate the common practice of pre-testing the PH assumption. Instead, we suggest directly applying a procedure which can detect survival curve differences in PH as well as non-PH settings, such as the methods presented in this paper.
2. the quality of the data presentation and the report of all relevant information. This includes, in particular, the table of the number at risks at multiple time points, which was not reported in almost 30% of the reviewed publications. These tables and all relevant information can be easily accessed through each common statistical software and should be provided in every study report. They are mandatory for a reliable assessment of the results and, moreover, facilitate a secondary analysis, e.g. for meta-analysis studies, by reconstructing the original data in a reasonable quality [25].

## Conclusion

We conclude that in case of non-PH, the choice of a suitable test procedure is relevant and the LR is not always the best choice. Therefore, we recommend to use all prior information available and to consider more options to test for differences in survival than just the LR. In terms of study design there are still some limitations since not all of the tests are used for sample size estimation and some tests are not freely available in R (see the Supplements for more information). Finally, we recommend using omnibus tests such as the mdir test for inference when no prior information on the pattern of hazards is available.

## Supplementary Information

**Additional file 1.**

## Data Availability

The datasets used and/or analyzed during the current study are available from the corresponding author on reasonable request.

## Abbreviations

2ST: Two-stage test
ABC: Area between curves
coxRMST: Combined Cox and permutation based RMST test
IPD: Individual patient data
KM: Kaplan-Meier
KONP chi: K-sample omnibus non-proportional hazards test with chi-square test statistic
KONP llr: K-sample omnibus non-proportional hazards test with log likelihood ratio type test statistic
LR: Log-rank test
mdir: Multiple-direction log-rank test
PH: Proportional hazards
PP: Peto-Peto test
RMST: Restricted mean suvival time
