# Supplementary material for: Which test for crossing survival curves? A user’s guideline
Source: BMC Med Res Methodol. 2022 Jan 30;22:34. doi: 10.1186/s12874-022-01520-0 (PMC8802494; doi:10.1186/s12874-022-01520-0)
Supplement: Supplementary file 1 — Additional file 1. [file 12874_2022_1520_MOESM1_ESM.docx]

**1 Detailed Information**

## Log-rank test

The log-rank test is the most applied test for an overall comparison of two Kaplan-Meier curves of interest.^1^ It is based on the intuitive comparison of observed and expected values.^2^ The test has optimal power in case of PH but can lead to poor rejection rates when the survival curves cross. By adding a weight function, we can obtain a test being more powerful for other kind of hazard alternatives, e.g. early, late or even crossing hazard differences.^3^ A weighted version of the log-rank test statistic is given by

$Z_{n}\left( w \right)= \frac{\sum_{i=1}^{D} w_{i}(N_{i1}-Y_{i1}\frac{N_{i}}{Y_{i}})}{\sqrt{\sum_{i=1}^{D} w_{i}^{2}\frac{Y_{i1}}{Yi}\left( 1- \frac{Y_{i1}}{Y_{i}} \right)\frac{Y_{i}-N_{i}}{Y_{i}-1}N_{i}}}$ *.* (1)

Here, $N_{ij}$ denotes the number of events in group $j=1,2$at event time $t_{i}, i=1,\ldots,D$and $Y_{ij}$ the corresponding number at risk.^4^  $Y_{i}$and $N_{i}$are the number at risk and number of events in the pooled sample. Setting $w_{i}\equiv1$gives the well-known log-rank (LR) statistic. Under $H_{0}$it follows the standard normal distribution. The log-rank test can be conducted by any common statistical software, e.g. by using the function *survdiff* from the *R* package *survival*. Many approaches to calculate sample sizes have been introduced for the log-rank test, the most referred to being Schoenfeld’s formula. ^5^ Another test within this general class of weighted log-rank tests is the Peto-Peto test introduced below.

## Peto-Peto test

The Peto-Peto test is a weighted log-rank test giving more emphasis to the early times.^6^ This is achieved by setting the weight $w_{i}$ in (1) equal to the survival estimate ${s(t}_{i})$ of the pooled sample at the event time $t_{i}$*.*The resulting test statistic follows, as the LR, a standard normal distribution under the null hypothesis. The Peto-Peto test as well as various other weighted log-rank tests are also implemented in the function *survdiff* from the *R* package *survival*. As the potential hazard difference (early, late, crossing etc.) is usually unclear, the Peto-Peto test as well as any other weighted log-rank test do not come to fore.

## mdir test

Recent advances, however, tackle the aforementioned problem by a flexible combination of several weighted log-rank tests into one testing procedure.^7–9^ It has lately been implemented in the R package *mdir.logrank* ^10^. Without any prior knowledge, the authors of the package recommend a combination of the classical log-rank weight $w_{i}^{(1)}\equiv1$ and a crossing weight$w_{i}^{(2)}\equiv1-2\hat{S}(t_{i})$, where $\hat{S}$ denotes the Kaplan-Meier estimator of the pooled sample. For this choice, the proposed test statistic is defined as the following studentized quadratic form

$$S_{n}=\left( Z_{n}\left( w^{\left( 1 \right)} \right), Z_{n}\left( w^{\left( 2 \right)} \right) \right)\hat{\Sigma}_{n}^{-}\left( Z_{n}\left( w^{\left( 1 \right)} \right), Z_{n}\left( w^{\left( 2 \right)} \right) \right){}^{T},$$

where $\hat{\Sigma}_{n}^{-}$denotes the Moore–Penrose inverse of the empirical covariance matrix of $\left( Z_{n}\left( w^{\left( 1 \right)} \right), Z_{n}\left( w^{\left( 2 \right)} \right) \right).$This test statistic follows a *χ*^2^-distribution under $H_{0}$.^8^ For small sample sizes, a permutation approach is recommended.^7,8^ A version of the mdir test for K samples and factorial designs has been introduced by Ditzhaus et al.^11^.

## Max Combo test

Employing the maximum of the standardized weighted log-rank tests as a test statistic, the MaxCombo tests offers an alternative approach of combining multiple weights. The weight functions under consideration are of the Flemming-Harrington type.^12^ Thus, the test statistic can be given as

$$Z_{max}={max}_{\rho,\gamma}\left( Z_{FH\left( \rho,\gamma\right)} \right),$$

with $Z_{FH(\rho,\gamma)}$the standardized weighted log-rank test statistics. The documentation of the R package nphsim^13^ gives an example for possible weights. Critical values of the MaxCombo test can be derived based on asymptotic normality. Roychoudhury et al.^14^ present an iterative method to determine the sample size for analyses employing the maxCombo test. The two sample test is implemented in the R package nphsim^13^.

## Two-stage test

Qiu and Sheng^15^ introduce a two-stage method with reliable power in cases of proportional hazards and non-proportional hazards. In the first stage, the log-rank test is applied. When the null hypothesis can not be rejected, a test being sensitive for crossing hazards is performed in the second stage. This second test is built up by a weighted log-rank test with weights given by $w_{i}^{(m)}\equiv-1$ for all $i\leq m$and $w_{i}^{(m)}\equiv c_{m}$ for $i>m.$ The positive constant$c_{m}$ is adaptively calibrated such that the resulting weighted log-rank test $S_{n}\left( w^{\left( m \right)} \right)$is asymptotically independent to the classical log-rank test. The final test statistic for the second stage is the supremum of all weighted log-rank tests with crossing times $D_{\varepsilon}\leq m\leq D-D_{\varepsilon}$:

$V= \sup_{D_{\varepsilon}\leq m\leq D-D_{\varepsilon}} S_{n}(w^{(m)})$*,*

where $\varepsilon>0$ is a small number and $D_{\varepsilon}$equals the integer part of $D\varepsilon$. To ensure the type-I error control, the tests of both stages are conducted at a reduced

nominal level$\alpha_{1}=\alpha_{2}=1- \sqrt{1-\alpha}$. The p-values for the first and second tests, $p_{1}$and $p_{2}$, can be determined using the standard normal distribution and bootstrap respectively. The overall p-value is then given by

$$p=\left\{ \begin{aligned} p_{1}, if p_{1}\leq\alpha_{1} \\ \alpha_{1}+p_{2}\left( 1-\alpha_{1} \right), otherwise \end{aligned} \right.$$

and is, thus, adjusted in order to be compared with the overall significance level $\alpha$. The procedure is implemented in the *R* package *TSHRC*.^16^ Chen et al.^17^ proposed an improved version of the two-stage test which can be extended to the K sample case.

## RMST-based test

Beside HR, restricted mean survival times (RMST) can also be used to quantify the difference between two survival curves.^18^ It describes the mean event-free survival time up to a pre-defined time point $\tau$. According to Tian et al.^19^ the group difference in RMST can be estimated by the difference of the areas under the two Kaplan-Meier (KM) curves for a time up to $\tau.$

$$\hat{\Delta}\left( \tau\right)=\int_{0}^{\tau} \hat{S}_{1}(u)- \hat{S}_{2}\left( u \right)du.$$

There exist several implementations of RMST-based tests. The *R* package *surv2sampleComp* ^20^ uses a resampling method to obtain the p-value (RMST1) while the function *rmst2* in the package *survRM2* ^21^ uses asymptotic to obtain critical value (RMST2) (See Uno et al.^22^). Royston and Parmar^23^ introduced a test combining the log-rank test with a permutation-based RMST test (coxRMST).

The test was performed in Stata using the *stctest* command with default settings and a jackknife approach to determine the RMST.^24^ Royston ^25^ also provided an approach for sample size and power calculations for their coxRMST test. All of the RMST based tests are implemented for two group comparisons.

## ABC-based test

A disadvantage of RMST-based methods, which is often ignored, is that they just check for equality of the groups’ RMST values and, thus, are not specially designed to detect crossing survival curve departure. In particular, it is easy to define two crossing survival curves with the same RMST. To tackle this problem, Liu et al.^26^ proposed to consider the area between the curves (ABC) given by

$$T_{n}= \sqrt{n}\int_{0}^{\tau} |\hat{S}_{1}\left( u \right)-\hat{S}_{2}\left( u \right)|du$$

over a clinically relevant time window $[0,\tau]$. The authors explain that the critical value cannot be obtained via a standard normal approximation, as originally proposed by Lin and Xu^27^, but via a group-wise bootstrap. The method is not implemented in *R* yet, but the code is provided by the authors on request and is also added to the online supplement.

## KONP test

Gorfine et al.^28^ introduced an omnibus approach to address the nonproportional hazard problem for K-samples. For that purpose, they adopt the sample-space partition-based test of Heller et al.^29^ for uncensored observations to right-censored survival data. Roughly speaking, the idea is to consider $N$partitions of the two groups, each leading to a $2\times2$-contingency table. Thereby, the number $N$ of partitions depends on the data and especially on the censoring pattern. Then a summary statistic, e.g., the Pearson chi-squared or log-likelihood ratio statistic, is determined for each contingency table. The mean over all $N$summary statistics leads to the final test statistic. In order to obtain the corresponding p-value, the permutation imputation strategy of Wang et al.^30^ is adopted. The test for both aforementioned summary statistics is implemented in the *R* package *KONPsurv* ^31^.

| Method | R package | p-value calculation | Recommended number of samples for resampling | K- sample extension |
| --- | --- | --- | --- | --- |
| Log-rank | survival^32^ | asymptotic | - | yes |
| Peto-Peto | survival^32^ | asymptotic | - | yes |
| mdir | mdir.logrank^10^ | Asymptotic or bootstrap | 10000^8^ | Presented in Ditzhaus et al. ^11^ |
| MaxCombo | nphsim^13^ | asymptotic | - | no |
| Two-stage test | TSHRC^16^ | asymptotic or bootstrap | 1000^15^ | Presented in Chen et al.^17^ |
| RMST1 test | Surv2sampleComp^20^ | resampling | 1000^20^ | no |
| RMST2 test | survRM2^21^ | asymptotic | - | no |
| coxRMST test | Only available in Stat | permutation | 5000^24^ | no |
| ABC test | Supplement | Group-wise bootstrap | 2000^26^ | no |
| KONP test | KONPsurv^31^ | permutation | 1000^28^ | yes |

eTable 1: Overview of properties of the different methods

# Data reconstruction from Kaplan-Meier curves

Individual patient data from clinical studies is rarely available for reanalysis or meta-analysis. Guyot et al.^33^ present an algorithm to reconstruct individual patient data from Kaplan-Meier curves. Their approach uses the published curves and the total number of events, such as the number at risk at different time points, if available. To extract the KM coordinates from the curves, software such as WebPlotDigitizer^34^ can be used. The main idea is to consider intervals restricted by the time points with a given number at risk. Then, for each interval the number of events and censoring is computed iteratively until the estimated number at risk for the following interval equals the observed one. After iterating through all intervals, the estimated sum of events is compared to the published number of total events. In case of inequality the procedure is repeated until equality is obtained. In situations where no number of total events is given, this last re-adjustment cannot be performed. When only the number at risk at the start of the study is given, only one time interval is considered and the total number censored is assumed to be zero. The authors claim and illustrate by discussing several examples where lower quality of the results are produced due to less information available or low quality of the KM curves.

# 2 Documentation of the Screening of the Publications

| Criteria | Absolute | Relative |
| --- | --- | --- |
| KM curves not reported | 711 | 51% |
| More or less than two groups | 182 | 13% |
| No crossing or more than two crossings | 338 | 24% |
| Log-rank test significant | 37 | 3% |
| Number at risk not reported | 41 | 3% |
| bad quality of the curves | 10 | 1% |
| time-dependent censoring | 11 | 1% |
| small number of events | 13 | 1% |
| No logrank test | 39 | 3% |
| Total of excluded papers | 1382 | 99% |
| Reconstructed | 15 | 1% |
| Number at risk not reported (overall) | 197 | 14% (among reported KM-curves: 29%) |

eTable 2: Detailed documentation of the Screening process of 1400 papers. The total number of papers does not add up to 1400 in this table because some of the reconstructed papers appeared multiple times on PubMed and were not counted twice.

**3 Proportional hazards settings**

To evaluate the power of the tests under proportional hazard, we determined the performance the tests standard data sets available in R.^32,35^ The data sets were included if the score test (cox.zph from the survival^32^ in R) could not reject the null hypothesis of proportional hazards and if the log-rank test detects a difference in survival. The first data set considered is the lung data set^32^. We focus on the relationship between survival and sex and between survival and ECOG performance score (ph.ecog). Here, we only included the groups 1 and 2 in order to have a two-group comparison. The whas500 data set which considers several factors, such as gender and the survival time after heart attack.. The last data set studied in this context is the rats data set.^32^ For this analysis, we test for difference in survival between the different genders. eFigure 1 shows the log-log KM survival estimates on a log-time scale. Overall, the curves of all four subgroups do not strong evidence against PH. The majority of presented procedures rejects the respective null hypothesis (eTable 3), indicating good consistency under PH settings. The only exceptions are the two KONP tests when checking for the influence of the ECOG performance score on the survival in the lung cancer data sets at a significance level of 0.05.


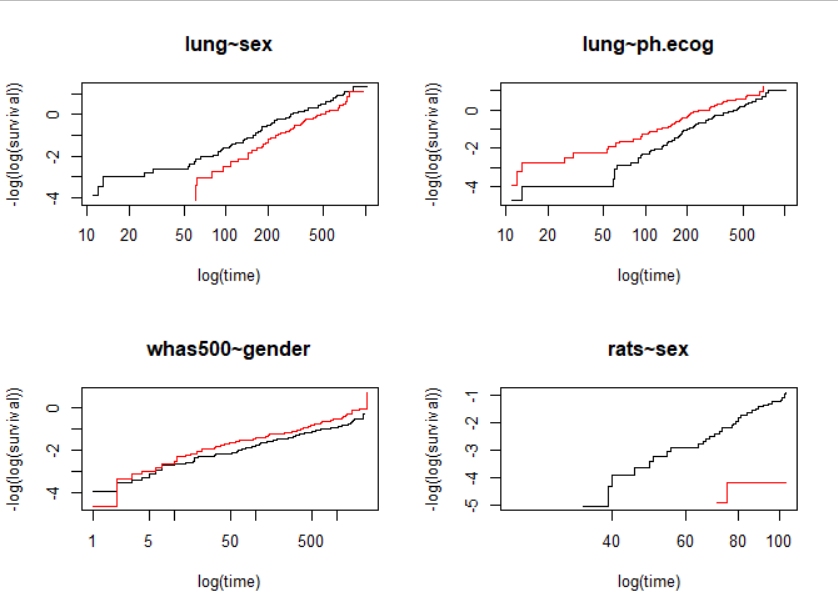


eFigure 1: Plot of log-log Kaplan Meier survival estimates the log of time

| data set ~ group | Score test | LR | PP | RMST1 | RMST2 | coxRMST | KONP_chi | KONP_llr | Mdir | 2ST | ABC | MaxCombo |
| --- | --- | --- | --- | --- | --- | --- | --- | --- | --- | --- | --- | --- |
| lung ~ sex | 0.09 | 0.001 | <0.001 | <0.001 | <0.001 | 0.002 | 0.004 | 0.004 | 0.001 | 0.001 | <0.001 | <0.001 |
| lung ~ ph.ecog | 0.17 | 0.004 | 0.001 | <0.001 | <0.001 | 0.002 | 0.051 | 0.054 | 0.007 | 0.001 | <0.001 | 0.014 |
| whas500 ~ gender | 0.38 | 0.005 | 0.009 | 0.005 | 0.005 | 0.008 | 0.013 | 0.013 | 0.011 | 0.005 | 0.007 | 0.021 |
| rats ~ sex | 0.46 | <0.001 | <0.001 | 0.004 | 0.004 | <0.001 | 0.003 | 0.001 | <0.001 | <0.001 | 0.004 | <0.001 |

eTable 3: P-values of the different tests applied to standard data sets in R with no rejected PH assumption

**4 Code**

**4.1 Minimum Example on how to apply the tests**

###########################################################################
# Applying the presented methods in R #
###########################################################################

##################################
# Load data
##################################

# #install a required package (only needed the first time)
#install.packages("coxphw")
# Load the package (always)
library(coxphw)

# Load the data from the package
data("gastric")
# Some tests require groups coded as 0 and 1:
gastric$group[gastric$group == 2] <-0

# Significance level
alpha <- 0.05


##################################
# Log-rank test
##################################
# Load required package
#install.packages("survival")
library(survival)

# Execute test
logRank = survdiff(Surv(time, status)~ group, rho = 0,
 #rho indicates classical log-rank test
 data = gastric)$chisq
1 - pchisq(logRank,1)

##################################
# Peto-Peto test
##################################
# Load required package
#install.packages("survival")
library(survival)

# Execute test
PetoPeto = survdiff(Surv(time, status)~ group, rho = 1,
 # Peto & Peto modification
 data = gastric)$chisq
1 - pchisq(PetoPeto,1)


##################################
# Gorfine et al. test
##################################
# Load required package
#install.packages("KONPsurv")
library(KONPsurv)

# Define parameters
iterations <- 999

# Execute test
KONP = konp_test(gastric$time, gastric$status, gastric$group,
 # as input wee need time, censoring indicator and group
 n_perm = iterations)
 # and number of iterations for permutation
# The two implemented test statistics
KONP$pv_chisq
KONP$pv_lr

##################################
# Ditzhaus and Friedrich test
##################################
# Load required package
#install.packages("mdir.logrank")
library(mdir.logrank)

# Define parameters
iterations <- 999

# Prepare data
gastric_mdir <- gastric
colnames(gastric_mdir) <- c("time", "event", "group")
# colnames as required for D&F

# Execute test
mdir.logrank(gastric_mdir, cross =TRUE, #include crossing alternative
 nperm = iterations)$p_value$Perm

##################################
# Tian et al. test
##################################
# Load required package
#install.packages("surv2sampleComp")
library(surv2sampleComp)

# Define parameters
iterations <- 999
tau <- 2900

# Execute test
RMST1 = surv2sample(gastric$time, gastric$status, gastric$group,
 npert = iterations,
 timepoints = tau,
 # when to calculate difference and ratio
 tau = tau, # restricted timepoint
 conf.int = 1-alpha)$integrated_surv.diff
RMST1[1,4] # p-value

##################################
# Uno et al. test
##################################
# Load required package
#install.packages("survRM2")
library(survRM2)


# Define parameters
iterations <- 999
tau <- 2900

# Execute test
RMST2 = rmst2(gastric$time, gastric$status, gastric$group,
 tau, # restricted timepoint
 covariates = NULL, alpha)
RMST2$unadjusted.result[1,4] # p-value

##################################
# Sheng et al. test
##################################
# Load required package
#install.packages("TSHRC")
library(TSHRC)

# Define parameters
iterations <- 999

# Execute test
twostage(gastric$time, gastric$status, gastric$group,
 nboot=iterations)[3]

##################################
# Plot
##################################

install.packages("survminer")
library(survminer)

# Create a survival curve
fit <- survfit(Surv(gastric$time, gastric$status)~gastric$group,
 # create survival object from time and status with
 # arms according to group
 data = gastric, type = "kaplan-meier")

# Visualize with survminer
ggsurvplot(fit, data = gastric, risk.table = TRUE,
 # we do want a risk table
 break.time.by = 500, # where to break the x axis
 break.y.by = 0.1, # where to break the y axis
 xlim = c(0,3000), ylim = c(0,1), xlab = "\n Months"
 , ylab = "\n",
 legend.labs = c("chemotherapy alone",
 "chemotherapy plus radiation"),
 palette = c("#0073b5", "#e39144"), # Define colors
 legend = c(0.7, 0.7), # position of the legend
 legend.title="", fontsize = 5, # fontsize for the table
 font.x = c(14, "bold"), font.caption = c(14, "black"),
 font.legend = c(14, "black"), font.tickslab = c(14, "black"),
 font.table = c(14, "black"),
 tables.theme = theme_cleantable(),
 # Minimalistic style of the table
 tables.height = 0.2, # change hight of the table
 risk.table.y.text = FALSE, # no group names in the table
 tables.y.text = FALSE)

**4.2 The ABC test**

### Code for P1: Alternatives to the hazard ratio -
### a simulation-based review

## Part III: A resampling-based test for two crossing survival curves
hatS=function(sample,Time){
 # Return Kaplan-Meiter estimator at t=Time

 # input: sample: a observed survival sample (X,delat), where X=min(T,C).
 # Time£ºThe time points at which that we want to estiamte,
 # can be avector or a signel time point.

 surFit=survfit(Surv(sample[,1],sample[,2])~1,error="greenwood")
 surEst=stepfun(surFit$time,c(1,surFit$surv))

 # Estimate
 hatSKM=surEst(Time)
 return(hatSKM)
}

hatGamma=function(t,deltaN1,deltaN2,Y1,Y2,index1,index2,n){
 ## Compute hatGamma(t)
 ## input: t is the given time
 ## deltaN1: Number of failure at failure time point of sample 1
 ## deltaN2: Number of failure at failure time point of sample 2
 ## Y1 : Number of at risk at failure time point of sample 1
 ## Y2 : Number of at risk at failure time point of sample 2
 ## index1 : Failure time points of sample 1 less or equal to t
 ## index2 : Failure time points of sample 2 less or equal to t

 ## Gamma1t=sum_{t_i<=t}[DeltaN1(t_i)/{(Y1(t_i)-DeltaN1(t_i))*Y1(t_i)}]
 gamma1t=deltaN1[index1]/((Y1[index1]-deltaN1[index1])*Y1[index1])
 ## Gamma2t=sum_{t_i<=t}[DeltaN2(t_i)/{(Y2(t_i)-DeltaN2(t_i))*Y2(t_i)}]
 gamma2t=deltaN2[index2]/((Y2[index2]-deltaN2[index2])*Y2[index2])
 ## Adjust the infinity value to 0
 gamma1t[gamma1t==Inf]=0
 gamma2t[gamma2t==Inf]=0

 hatGamma_t=n*(sum(gamma1t)+sum(gamma2t))
 return(hatGamma_t)}

library(MASS)
scaledABC=function(sample1,sample2,alpha)
{ # return test statistics T_n, upper alpha quantile
 # of its bootstrap distribution,
 # and upper alpha quantile of its asymptotic distribution

 # input:
 # sample1: survival data of sample 1
 # sample2: survival data of sample 2
 # alpha : the significant level

 n1=dim(sample1)[1];n2=dim(sample2)[1];n=n1+n2

 K=9.4

 # Failure time points for sample 1, sample2, and pooled samples
 failureTime1=sort(sample1[sample1[,2]==1,1])
 failureTime2=sort(sample2[sample2[,2]==1,1])
 pooledSample=rbind(sample1, sample2)
 pooledFailureTime=sort(pooledSample[pooledSample[,2]==1,1])
 pooledFailureTime = pooledFailureTime[pooledFailureTime < K]
 numPooledFailure=length(pooledFailureTime)


 # fit survival curves by KM
 sur.fit1=survfit(Surv(sample1[,1],sample1[,2])~1,error="greenwood")
 sur.fit2=survfit(Surv(sample2[,1],sample2[,2])~1,error="greenwood")

 # create KM estimators by step function
 sur.est1=stepfun(sur.fit1$time,c(1,sur.fit1$surv))
 sur.est2=stepfun(sur.fit2$time,c(1,sur.fit2$surv))

 # Estimate S_j(t) at failure time points
 S1=sur.est1(pooledFailureTime);
 S2=sur.est2(pooledFailureTime);

 # gap time t_{i+1}-t_{i}
 gapTime=diff(c(pooledFailureTime,K))

 # T_n=sqrt(n)*sum_{i=1}^{kn}|s1(t_i)-s2(t_i)|*(t_{i+1}-t_i)
 Tn=sqrt(n)*sum(abs(S1-S2)*gapTime)

 # quantile of Tn by bootstrapping
 B=2000; bootTn=rep(0,B)

 for(iB in 1:B){
 # make the bootstrap procedure replicable
 set.seed(iB)
 # Bootstrap sample 1
 bootSample1=sample1[sample(n1,replace = TRUE),]

 set.seed(-iB)
 # Bootstrap sample 2
 bootSample2=sample2[sample(n2,replace = TRUE),]

 # Tn*=sqrt(n)*sum_{i=1}^{kn}|{S1*(t_i)-S1(t_i)}
 # -{S2*(t_i)-S2(t_i)}|*(t_{i+1}-t_i)
 bootTn[iB]=sqrt(n)*sum(abs( (hatS(bootSample1,pooledFailureTime)-S1)
 -(hatS(bootSample2,pooledFailureTime)-S2) )
 *gapTime)
 } # end of for boot
 quantileBootTn=quantile(bootTn,(1-alpha))

 return(res=list(Tn=Tn,quantileBootTn=quantileBootTn,
 bootP=mean(bootTn>=Tn)))
}

**References**

1. Singh R, Mukhopadhyay K. Survival analysis in clinical trials: Basics and must know areas. *Perspect Clin Res*. 2011;2(4):145.

2. Kleinbaum DG, Klein M. *Survival Analysis*. Vol 3. Springer; 2010.

3. Fleming TR, Harrington DP. *Counting Processes and Survival Analysis*. John Wiley & Sons; 2011.

4. Klein JP, Moeschberger ML. *Survival Analysis: Techniques for Censored and Truncated Data*. 2nd ed. Springer; 2003.

5. Schoenfeld DA. Sample-Size Formula for the Proportional-Hazards Regression Model. *Biometrics*. 1983;39(2):499. doi:10.2307/2531021

6. Legrand C. *Advanced Survival Models*. CRC Press; 2021.

7. Brendel M, Janssen A, Mayer CD, Pauly M. Weighted Logrank Permutation Tests for Randomly Right Censored Life Science Data: Weighted logrank permutation tests. *Scand J Stat*. 2014;41(3):742-761. doi:10.1111/sjos.12059

8. Ditzhaus M, Friedrich S. More powerful logrank permutation tests for two-sample survival data. *ArXiv180705504 Math Stat*. Published online July 15, 2018. Accessed May 6, 2020. http://arxiv.org/abs/1807.05504

9. Ditzhaus M, Pauly M. Wild bootstrap logrank tests with broader power functions for testing superiority. *Comput Stat Data Anal*. 2019;136:1-11.

10. Ditzhaus M, Friedrich S. *Mdir.Logrank: Multiple-Direction Logrank Test*.; 2018. Accessed October 25, 2021. https://CRAN.R-project.org/package=mdir.logrank

11. Ditzhaus M, Genuneit J, Janssen A, Pauly M. CASANOVA: Permutation inference in factorial survival designs. *Biometrics*. Published online October 5, 2021:biom.13575. doi:10.1111/biom.13575

12. Lee SH. On the versatility of the combination of the weighted log-rank statistics. *Comput Stat Data Anal*. 2007;51(12):6557-6564.

13. Wang Y, Wu H, Anderson KM, Roychoudhury S, Hu T, Liu H. *Nphsim: Non Proportional Hazards Sample Size and Simulation*.

14. Roychoudhury S, Anderson KM, Ye J, Mukhopadhyay P. Robust Design and Analysis of Clinical Trials With Nonproportional Hazards: A Straw Man Guidance From a Cross-Pharma Working Group. *Stat Biopharm Res*. Published online 2021:1-15.

15. Qiu P, Sheng J. A two‐stage procedure for comparing hazard rate functions. *J R Stat Soc Ser B Stat Methodol*. 2008;70(1):191-208.

16. Sheng J, Qiu P, Geyer and CJ. *TSHRC: Two Stage Hazard Rate Comparison*.; 2019. Accessed October 25, 2021. https://CRAN.R-project.org/package=TSHRC

17. Chen Z, Huang H, Qiu P. An improved two-stage procedure to compare hazard curves. *J Stat Comput Simul*. 2017;87(9):1877-1886.

18. Kim DH, Uno H, Wei LJ. Restricted mean survival time as a measure to interpret clinical trial results. *JAMA Cardiol*. 2017;2(11):1179-1180.

19. Tian L, Fu H, Ruberg SJ, Uno H, Wei LJ. Efficiency of two sample tests via the restricted mean survival time for analyzing event time observations: Efficiency of Two Sample Tests via the Restricted Mean Survival Time. *Biometrics*. 2018;74(2):694-702. doi:10.1111/biom.12770

20. Tian L, Uno H, Horiguchi M. *Surv2sampleComp: Inference for Model-Free Between-Group Parameters for Censored Survival Data*. Accessed October 25, 2021. https://rdrr.io/cran/surv2sampleComp/man/surv2sample.html

21. Uno H, Tian L, Horiguchi M, Cronin A, Battioui C, Bell J. *SurvRM2: Comparing Restricted Mean Survival Time*.; 2020. Accessed October 25, 2021. https://CRAN.R-project.org/package=survRM2

22. Uno H, Claggett B, Tian L, et al. Moving Beyond the Hazard Ratio in Quantifying the Between-Group Difference in Survival Analysis. *J Clin Oncol*. 2014;32(22):2380-2385. doi:10.1200/JCO.2014.55.2208

23. Royston P, Parmar MKB. Augmenting the logrank test in the design of clinical trials in which non-proportional hazards of the treatment effect may be anticipated. *BMC Med Res Methodol*. 2016;16(1):16. doi:10.1186/s12874-016-0110-x

24. Royston P. A Combined Test for a Generalized Treatment Effect in Clinical Trials with a Time-to-event Outcome. *Stata J Promot Commun Stat Stata*. 2017;17(2):405-421. doi:10.1177/1536867X1701700209

25. Royston P. Power and sample-size analysis for the Royston-Parmar combined test in clinical trials with a time-to-event outcome. *Stata J*. 18:3-21.

26. Liu T, Ditzhaus M, Xu J. A resampling‐based test for two crossing survival curves. *Pharm Stat*. Published online 2020.

27. Lin X, Xu Q. A new method for the comparison of survival distributions. *Pharm Stat J Appl Stat Pharm Ind*. 2010;9(1):67-76.

28. Gorfine M, Schlesinger M, Hsu L. K-sample omnibus non-proportional hazards tests based on right-censored data. *ArXiv Prepr ArXiv190105739*. Published online 2019.

29. Heller R, Heller Y, Gorfine M. A consistent multivariate test of association based on ranks of distances. *Biometrika*. 2013;100(2):503-510.

30. Wang R, Lagakos SW, Gray RJ. Testing and interval estimation for two-sample survival comparisons with small sample sizes and unequal censoring. *Biostatistics*. 2010;11(4):676-692.

31. Schlesinger M, Gorfine M. *KONPsurv: KONP Tests: Powerful K-Sample Tests for Right-Censored Data*.; 2020. Accessed October 25, 2021. https://CRAN.R-project.org/package=KONPsurv

32. Therneau TM, until 2009) TL (original S >R port and R maintainer, Elizabeth A, Cynthia C. *Survival: Survival Analysis*.; 2021. Accessed November 1, 2021. https://CRAN.R-project.org/package=survival

33. Guyot P, Ades A, Ouwens MJ, Welton NJ. Enhanced secondary analysis of survival data: reconstructing the data from published Kaplan-Meier survival curves. *BMC Med Res Methodol*. 2012;12(1):9. doi:10.1186/1471-2288-12-9

34. WebPlotDigitizer - Extract data from plots, images, and maps. Accessed October 25, 2021. https://automeris.io/WebPlotDigitizer/

35. smoothHR: Smooth Hazard Ratio Curves Taking a Reference Value version 1.0.3 from CRAN. Accessed November 4, 2021. https://rdrr.io/cran/smoothHR/
